# Electron Bifurcation and Confurcation in Methanogenesis and Reverse Methanogenesis

**DOI:** 10.3389/fmicb.2018.01322

**Published:** 2018-06-20

**Authors:** Zhen Yan, James G. Ferry

**Affiliations:** Department of Biochemistry and Molecular Biology, Pennsylvania State University, State College, PA, United States

**Keywords:** archaea, heterodisulfide reductase, methane, ferredoxin, hydrogen, acetate, formate

## Abstract

Reduction of the disulfide of coenzyme M and coenzyme B (CoMS–SCoB) by heterodisulfide reductases (HdrED and HdrABC) is the final step in all methanogenic pathways. Flavin-based electron bifurcation (FBEB) by soluble HdrABC homologs play additional roles in driving essential endergonic reactions at the expense of the exergonic reduction of CoMS–SCoM. In the first step of the CO_2_ reduction pathway, HdrABC complexed with hydrogenase or formate dehydrogenase generates reduced ferredoxin (Fdx^2-^) for the endergonic reduction of CO_2_ coupled to the exergonic reduction of CoMS–SCoB dependent on FBEB of electrons from H_2_ or formate. Roles for HdrABC:hydrogenase complexes are also proposed for pathways wherein the methyl group of methanol is reduced to methane with electrons from H_2_. The HdrABC complexes catalyze FBEB-dependent oxidation of H_2_ for the endergonic reduction of Fdx driven by the exergonic reduction of CoMS–SCoB. The Fdx^2-^ supplies electrons for reduction of the methyl group to methane. In H_2_^-^ independent pathways, three-fourths of the methyl groups are oxidized producing Fdx^2-^ and reduced coenzyme F_420_ (F_420_H_2_). The F_420_H_2_ donates electrons for reduction of the remaining methyl groups to methane requiring transfer of electrons from Fdx^2-^ to F_420_. HdrA1B1C1 is proposed to catalyze FBEB-dependent oxidation of Fdx^2-^ for the endergonic reduction of F_420_ driven by the exergonic reduction of CoMS–SCoB. In H_2_^-^ independent acetotrophic pathways, the methyl group of acetate is reduced to methane with electrons derived from oxidation of the carbonyl group mediated by Fdx. Electron transport involves a membrane-bound complex (Rnf) that oxidizes Fdx^2-^ and generates a Na^+^ gradient driving ATP synthesis. It is postulated that F_420_ is reduced by Rnf requiring HdrA2B2C2 catalyzing FBEB-dependent oxidation of F_420_H_2_ for the endergonic reduction of Fdx driven by the exergonic reduction of CoMS–SCoB. The Fdx^2-^ is recycled by Rnf and HdrA2B2C2 thereby conserving energy. The HdrA2B2C2 is also proposed to play a role in Fe(III)-dependent reverse methanogenesis. A flavin-based electron confurcating (FBEC) HdrABC complex is proposed for nitrate-dependent reverse methanogenesis in which the oxidation of CoM-SH/CoB-SH and Fdx^2-^ is coupled to reduction of F_420_. The F_420_H_2_ donates electrons to a membrane complex that generates a proton gradient driving ATP synthesis.

## Introduction

**FIGURE 1:**
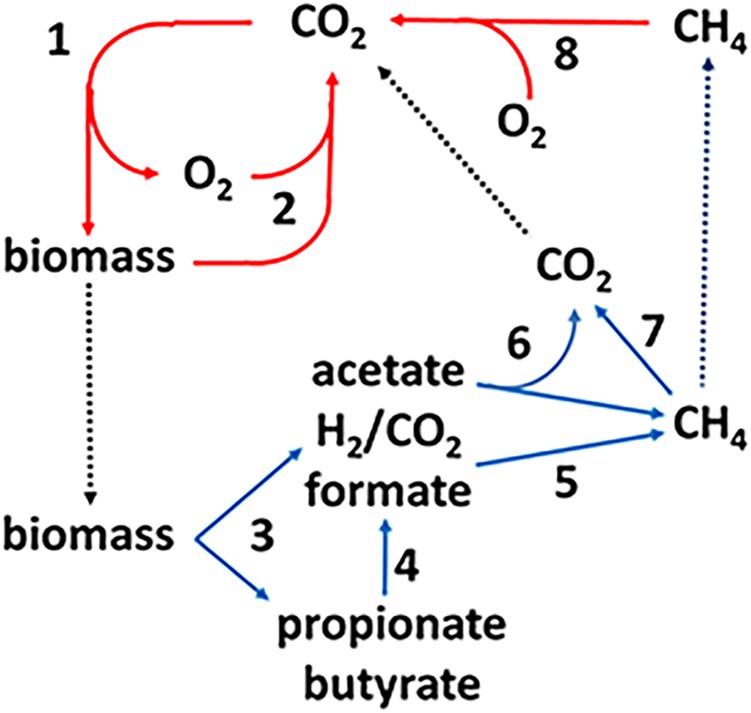
The global carbon cycle. Solid lines indicate aerobic (red) and anaerobic (blue) steps in the cycle and dotted lines indicate transfer of material between aerobic and anaerobic environments. (1) Photosynthesis, (2) aerobic decomposition, (3) fermentation, (4) syntrophic acetogenesis, (5) CO_2_ reduction to methane with electrons derived from formate or H_2_, (6) acetotrophic methanogenesis, (7) anerobic oxidation of methane, and (8) aerobic oxidation of methane.

Methane-producing archaea (methanogens) are terminal organisms of anaerobic microbial food chains decomposing complex organic matter in Earth’s anaerobic biosphere which includes the lower intestinal tract of humans, the hind gut of termites, the rumen of animals, natural wetlands and rice paddies. As such, methanogens are an essential link in the global carbon cycle (**Figure 1**). In step 1, CO_2_ is incorporated into biomass by photosynthetic plants and microbes. In oxygenated environments, O_2_-respiring microbes oxidize the biomass producing CO_2_ that re-enters the carbon cycle (step 2). A significant fraction of the biomass enters anaerobic biospheres where it is converted to CO_2_ and CH_4_ by microbial food chains comprised of at least four metabolic groups (steps 3–6). The fermentative group digests the complex biomass producing acetate, H_2_, and CO_2_ along with other volatile fatty acids (step 3) that are oxidized to acetate plus either formate or H_2_ (step 4) by syntrophic acetogens. The CO_2_-reducing methanogen group reduces CO_2_ to CH_4_ with electrons derived from oxidation of H_2_ or formate (step 5). This group forms symbioses with the acetogens that supply H_2_ or formate the methanogens metabolize to concentrations thermodynamically favorable for the acetogens in a process termed interspecies electron transfer (ISET) (Sieber et al., 2009). The acetate-utilizing (acetoclastic) methanogen group converts the methyl group to CH_4_ and the carbonyl group to CO_2_ (step 6). A portion of the CH_4_ is oxidized to CO_2_ (step 7) by the anaerobic oxidation of methane (AOM) proposed to involve the reversal of methanogenic pathways. The CO_2_ and remaining CH_4_ escapes into oxygenated zones where O_2_-respiring methanotrophic microbes oxidize CH_4_ to CO_2_ (step 8), closing the carbon cycle. As a greenhouse gas, methane is nearly 20-fold more potent than CO_2_; thus, the aerobic and anaerobic oxidation of CH_4_ plays an important role in controlling Earth’s climate (Valentine, 2002; Rhee et al., 2009).

Electron transport is much less understood than the comprehensive biochemical understanding of carbon transformations in methanogenic and reverse methanogenic pathways. Herein is reviewed the current understanding of electron transport with a focus on the role of flavin-based electron bifurcation (FBEB) and confurcation (FBEC).

## Obligate Co_2_ Reducing Methanogens

**FIGURE 2:**
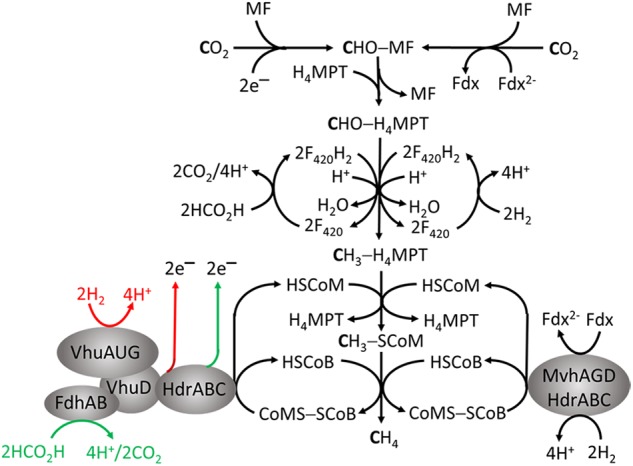
Roles for flavin-based electron bifurcation (FBEB) by heterodisulfide reductases in the formate- and H_2_-dependent CO_2_ reduction pathway of methanogenesis in *Methanothermobacter marburgensis* and *Methanococcus maripaludis*. The red and green arrows represent the FBEB-dependent generation of electrons by HdrABC originating from oxidation of H_2_ and HCO_2_H, respectively, for reduction of CO_2_ in the first step of the pathway.

As the name implies, this group only produces CH_4_ by reducing CO_2_, primarily with electrons from oxidation of H_2_ or formate. The pathway (**Figure 2**) is the subject of reviews (Liu and Whitman, 2008; Thauer et al., 2008; Ferry, 2010). The first step is reduction of CO_2_ to formyl-methanofuran (CHO-MF) catalyzed by formylmethanofuran dehydrogenase (Fwd or Fmd) (Wagner et al., 2016). The reaction is endergonic and dependent on reduced ferredoxin (Fdx^2-^). The formyl group of CHO-MF is transferred to tetrahydromethanopterin (H_4_MPT) and reduced to yield CH_3_ - H_4_MPT. Most methanogens contain H_4_MPT, whereas *Methanosarcina* species contain the functionally equivalent tetrahydrosarcinapterin (H_4_SPT). The electron donor is reduced coenzyme F_420_ (F_420_H_2_) generated from H_2_ or formate by F_420_-dependent hydrogenases (Fru, Frc, Frh) or formate dehydrogenase (Fdh) (Thauer et al., 2010). Coenzyme F_420_ is an obligate two-electron carrier donating or accepting a hydride. The methyl group of CH_3_ - H_4_MPT is transferred to coenzyme M (HS-CoM) catalyzed by methyltransferase (Mtr) to generate CH_3_ - SCoM. This exergonic reaction is linked to translocation of Na^+^ outside the membrane generating a gradient (high outside). Without cytochromes in obligate CO_2_ reducing methanogens, this is the only mechanism generating an ion gradient that drives ATP synthesis (Thauer et al., 2008). Methyl-SCoM methylreductase (Mcr) catalyzes the reductive demethylation of CH_3_ - SCoM to CH_4_ involving coenzyme B (HS-CoB) accompanied by formation of CoMS-SCoB. Heterodisulfide reductase (HdrABC) reduces the disulfide bond with electrons supplied from the oxidation of 2H_2_ or 2HCO_2_H (*E*^∘^′ ∼-420 mV) catalyzed by F_420_-independent hydrogenase or Fdh. The exergonic reduction of CoMS-SCoB (*E*^∘^′ = –140 mV) drives the endergonic reduction of CO_2_ (*E*^∘^′ = –500 mV) in the first step (**Figure 2**) via FBEB by HdrABC (Buckel and Thauer, 2013).

**FIGURE 3:**
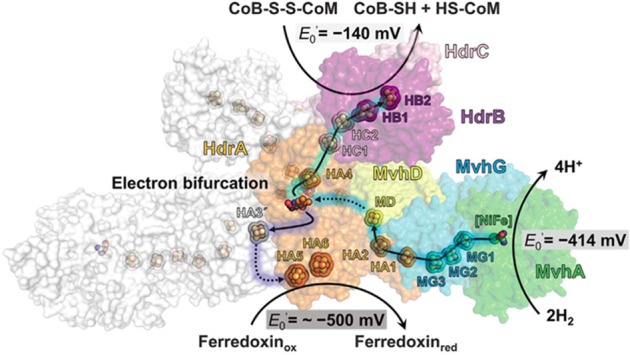
Proposed electron-transfer pathway in the heterodisulfide reductase [NiFe]–hydrogenase complex (HdrABC-MvhAGD) from *Methanothermococcus thermolithotrophicus*. MvhA (green), MvhG (cyan), MvhD (yellow), HdrA (orange), HdrB (purple), HdrC (light pink). The solid arrows indicate the electron-transfer pathway composed of iron-sulfur clusters at distances less than 13.5 Å. The dashed arrows correspond to a hypothetical electron-transfer pathway with distances longer than 15 Å between the redox centers. Reproduced by permission (Wagner et al., 2017).

**FIGURE 4:**
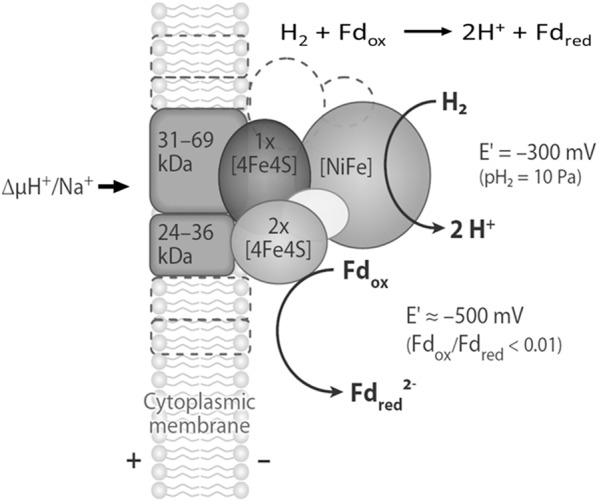
Schematic representation of the structure and function of the energy-converting [NiFe]-hydrogenases EchA-F, EhaA-T, EhbA-Q, and MbhA-N found in methanogenic archaea. The energy-converting hydrogenase EchA-F is composed only of the six conserved core subunits, which are shown in shades of gray. The energy-converting hydrogenases EhaA-T, EhbA-Q, and MbhA-N also contain several hydrophobic and hydrophilic subunits of unknown function. These subunits are symbolized by areas with dashed boundaries. Abbreviations: Fd_ox_, oxidized ferredoxin (Fdx); Fd_red_^2-^, two-electron-reduced ferredoxin (Fdx^2-^). Adapted from (Thauer et al., 2010).

Two variations are proposed for FBEB of obligate CO_2_-reducing methanogens (**Figure 2**). FBEB in strictly hydrogenotrophic *Methanothermobacter marburgensis* involves the soluble MvhAGD:HdrABC for which the crystal structure of the heterododecameric complex from *Methanothermococcus thermolithotrophicus* supports a proposed mechanism (**Figure 3**; Thauer et al., 2008; Kaster et al., 2011; Buckel and Thauer, 2013; Wagner et al., 2017). H_2_ is oxidized at the catalytic [NiFe] center of MvhA with transfer of electrons to the [2Fe-2S] cluster of MvhD mediated by the [4Fe-4S] clusters of MvhA and MvhG. The bifurcating FAD of HdrA sequentially accepts two electrons from the [2Fe-2S] cluster that contrasts with other characterized FBEB enzymes for which a hydride is donated to FAD (Lubner et al., 2017; Peters and Lubner, 2017). Any of three conformational changes are proposed to overcome the >30 Å distance observed in the crystal structure that otherwise would prohibit electron transfer between the [2Fe-2S] cluster of MvhD and FAD of HdrA. At this juncture the electrons from reduced FAD (FADH^-^) bifurcate into a high-potential and a low-potential electron. The high-potential electron from FADH^-^ is transported via [4Fe-4S] clusters of HdrA and HdrC to the active-site non-cubane [4Fe-4S] clusters of HdrB where CoMS-SCoB is reduced. The low-potential electron of the resulting semiquinone radical (FADH) is transported via [4Fe-4S] clusters (HA3, HA5, and HA6) of HdrA to Fdx. It is proposed that a conformational change overcomes the 21.5 Å distance in the crystal structure that would otherwise prohibit electron transfer between HA3 and HA5 (**Figure 3**). The structure reveals residues adjacent to the isoalloxazine ring of FAD proposed to achieve the low-potential neutral FADH radical and a postulated anionic semiquinone (FAD^-^) intermediate during reduction of FAD. The process occurs twice yielding HSCoM, HSCoB, and Fdx^2-^ that donates electrons to Fwd reducing CO_2_ to CHO-MF (**Figure 2**). Generation of Fdx^2-^ by the membrane-bound energy-converting hydrogenase Eha (**Figure 4**) of obligate CO_2_ reducing methanogens serves an anaplerotic role and validates the essentiality of FBEB (Lie et al., 2012).

In contrast to strictly hydrogenotrophic *M. marburgensis*, *Methanococcus maripaludis* utilizes either H_2_ or formate as electron donors for reduction of CO_2_ to CH_4_ requiring FBEB mechanisms for each substrate (**Figure 2**). A protein complex isolated from formate-grown cells contains HdrABC, F_420_-non-reducing selenocysteine-containing hydrogenase (Vhu), Fdh, and the tungsten-containing Fwd (Costa et al., 2010). This result lead to the conclusion that Fdh oxidizes formate with direct transfer of electrons to HdrABC without first producing H_2_ by a F_420_-dependent formic hydrogenlyase system and then oxidation of the H_2_ by the F_420_-independent MvhAGD hydrogenase as in FBEB by *M. marburgensis*. Direct transfer without participation of H_2_ as an intermediate is supported by robust growth with formate, although not H_2_, for a mutant deleted of genes encoding subunits of the selenocysteine-containing (vhu) and cysteine-containing (vhc) F_420_-independent hydrogenases associated with HdrABC. However, the mutant retained the vhuD and vhcD genes homologous to mvhD of *M. marburgensis* obscuring potential roles for VhuD and VhcD. When grown under conditions where both Fdh and Vhu are expressed, the enzymes compete for binding to VhuD, and are fully functional and bound to VhuD (Costa et al., 2013). Further, Fdh co-purifies with VhuD in the absence of other hydrogenase subunits. It was concluded that VhuD, also containing a [2Fe-2S] cluster, functions analogous to MvhD by mediating direct electron transfer from Vhu or Fdh to HdrABC (**Figure 2**; Costa et al., 2013). The mechanism for transfer of electrons from HdrABC to Fwd is unknown although likely mediated by Fdx as for *M. marburgensis* (Costa et al., 2010). Not reported is biochemical validation of electron bifurcation by the proposed complex as was shown for the MvhAGD:HdrABC and HdrA2B2C2 complexes of *M. marburgensis* and *Methanosarcina acetivorans* (Kaster et al., 2011; Yan et al., 2017). Nonetheless, an *in silico* genome-scale metabolic reconstruction of *M. maripaludis* indicates the organism is unable to grow without the energy-conserving complex (Richards et al., 2016).

**FIGURE 5:**
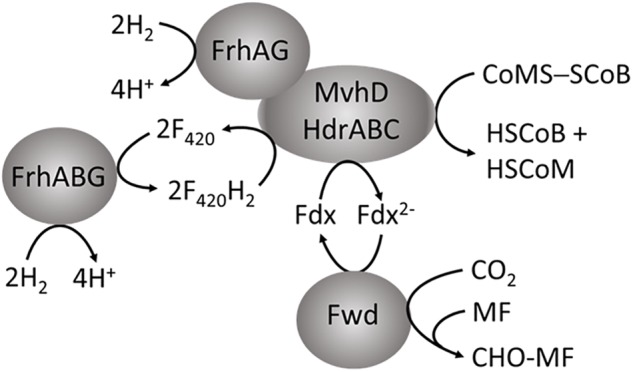
Proposed role for FBEB by the heterodisulfide reductase in obligate CO_2_-reducing methanogens of the order *Methanomicrobiales.* MF, methanofuran; Fdx, ferredoxin.

In addition to supplying Fdx^2-^ for the first step in the CO_2_-reduction pathway of methanogenesis, it is proposed that energy-conserving FBEB is instrumental for growth of *Methanocella conradii* when concentrations of H_2_ are exceptionally low (Liu et al., 2014). A transcription unit comprised of genes encoding Fwd, HdrABC and MvhD is up regulated in *M. conradii* grown syntrophically in co-culture with H_2_-producing acetogens utilizing propionate and butyrate (Liu et al., 2014). Thus, it is proposed that an electron bifurcating MvhD/HdrABC/Fwd complex is essential for syntrophic growth with low concentrations of H_2_. As *M. conradii* encodes MvhGA remote from the up regulated transcription unit, the mechanism by which H_2_ is oxidized and electrons transferred to HdrABC is unknown. Interestingly, obligate CO_2_-reducing methanogens of the order *Methanomicrobiales* are missing genes encoding MvhA and MvhG but encode MvhD and HdrABC (Browne et al., 2016). These methanogens could form an MvhD/HdrABC complex associated with energy-converting hydrogenases EchA–F, EhaA–T, or MbhA–N dependent on ion gradients to supply Fdx^2-^ for reduction of CO_2_ to CHO-MF, although reduction of CoMS-SCoB would be energy consuming. Thus, it is proposed that these methanogens substitute MvhA and MvhG with FrhA and FrhG of the F_420_-reducing hydrogenase (FrhABG) contained in all methanogens without cytochromes (Thauer et al., 2010; Gilmore et al., 2017). In this way, FrhAG would be present in an FrhAG/MvhD/HdrABC complex with the potential for FBEB of H_2_ that generates the Fdx^2-^ required for reduction of CO_2_ to CHO-MF (**Figure 5**) with the added advantage of conserving energy. In this scenario, the energy-converting hydrogenases play a role in only providing Fdx^2-^ for biosynthesis (Major et al., 2010). However, the possibility of an FrhABG/MvhD/HdrABC complex (**Figure 5**) cannot be ruled out at this juncture. Inclusion of the F_420_-binding FrhB subunit invokes electron transport dependent on FrhABG producing F_420_H_2_ for which the electron pair is bifurcated by MvhD/HdrABC reducing Fdx and CoMS-SCoB analogous to the HdrA2B2C2 of *M. acetivorans* (Vitt et al., 2014; Yan et al., 2017). However, it is unknown which FBEB pathway is physiologically relevant.

## Methylotrophic Methanogens

Methanogens from the order *Methanosarcinales* grow and produce CH_4_ with methyl-containing substrates (methanol, methylamines, and methyl sulfides) (Liu and Whitman, 2008). A few also grow by reducing CO_2_ with H_2_. Unlike obligate CO_2_ reducers, these methanogens contain cytochromes and generate a proton gradient dependent on electron transport involving hydrogenases (Thauer et al., 2008). Like obligate CO_2_ reducers, Fdx^2-^ is required to supply electrons to Fwd/Fmd catalyzing reduction of CO_2_ to CHO-MF; however, Fdx^2-^ is generated independent of FBEB by the membrane-bound energy-converting Ech hydrogenase driven by the proton gradient (**Figure 4**).

**FIGURE 6:**
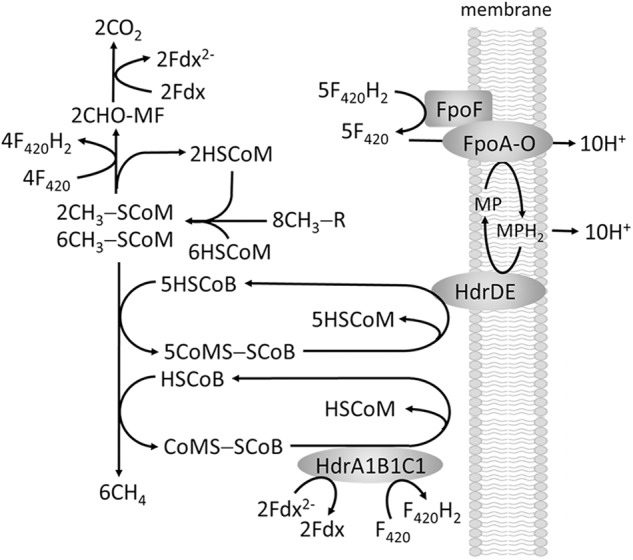
Proposed role of FBEC by HdrA1B1C1 in methylotrophic pathways of methanogenesis.

The methylotrophic pathway of the order *Methanosarcinales* involves transfer of substrate methyl groups to HSCoM forming a CH_3_-SCoM pool of which one-fourth of the methyl groups are oxidized to CO_2_ via reversal of the CO_2_-reduction pathway to supply F_420_H_2_ and Fdx^2-^ required for reductive demethylation of the remaining three-fourths CH_3_-SCoM to CH_4_ (**Figure 6**). The F_420_H_2_ is oxidized by a membrane-bound complex (Fpo) that donates electrons to a quinone-like electron carrier (methanophenazine, MP) coupled to generation of a proton gradient. It is proposed that Fdx^2-^ is re-oxidized by reducing F_420_ although the mechanism is unknown. The production of CH_4_ from CH_3_-SCoM is similar to obligate CO_2_ reducing methanogens involving HSCoB and Mcr with the exception of the membrane-bound heterodisulfide reductase (HdrDE) that reduces CoMS-SCoB to the sulfhydryl forms of the cofactors. Electrons are supplied to HdrDE by MPH_2_ with the scalar translocation of protons contributing to the proton gradient that drives ATP synthesis. However, the genomes of all sequenced *Methanosarcinales* also contain genes encoding the HdrABC homologs HdrA1B1C1 and HdrA2B2C2 (Buan and Metcalf, 2010). HdrA1B1C1 is elevated during methylotrophic growth of *M. acetivorans* for which the ΔhdrA1B1C1 mutant strain is growth impaired. Thus, a role in methylotrophic growth is proposed wherein FBEB by HdrA1B1C1 reduces F_420_ and CoMS-SCoB with electron pairs donated by two Fdx^2-^ generated in the oxidation of CHO-MF (**Figure 6**), thereby allowing energy conservation via Fpo (Buan and Metcalf, 2010).

**FIGURE 7:**
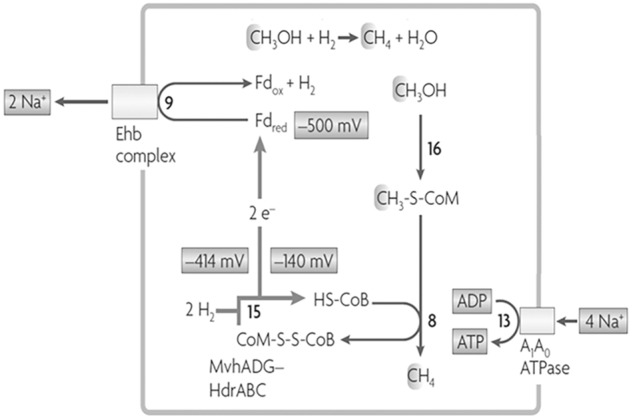
Proposed role for FBEB by heterodisulfide reductase in *Methanosphaera stadtmanae* growing with methanol and H_2_. Reactions 9 and 15 are coupled by FBEB. The redox potentials are standard potentials at pH 7.0. Fd_ox_, oxidized ferredoxin (Fdx); Fd_red_, two-electron-reduced ferredoxin (Fdx^2-^). Adapted from (Thauer et al., 2008).

**FIGURE 8:**
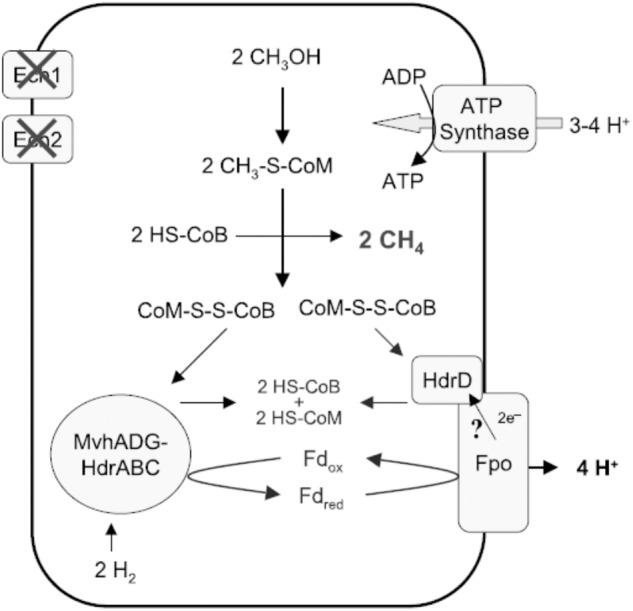
Proposed role for FBEB by heterodisulfide reductase in energy-conservation by *Methanomassiliicoccus luminyensis*. Ech1 and Ech2 are covered with crosses to indicate non-involvement in generating the proton gradient driving ATP synthesis (Kroninger et al., 2015). The question mark indicates a reaction not experimentally verified, although includes 2H^+^ pumped by the Fpo complex and another 2H^+^ translocated scalar via reduction and re-oxidation of methanophenazine. Fd_ox_, oxidized ferredoxin (Fdx); Fd_red_, two-electron-reduced ferredoxin (Fdx^2-^). Reproduced by permission (Kroninger et al., 2015).

*Methanosphaera stadtmanae*, isolated from the human gut, is also a methylotrophic methanogen reducing the methyl group of methanol to CH_4_ although belonging to the order *Methanobacteriales* that do not contain cytochromes necessary for electron-transport coupled proton translocation that drives ATP synthesis. The genome also lacks a complete gene set necessary for reversal of the CO_2_ reduction pathway and therefore requires the oxidation of H_2_ to supply electrons for reductive demethylation of CH_3_-SCoM to CH_4_ (Fricke et al., 2006). A scheme is proposed that includes FBEB of H_2_ by an MvhADG:HdrABC complex to explain the finding that ATP synthesis is driven by an ion gradient (**Figure 7**; Sparling et al., 1993; Thauer et al., 2008). The Fdx^2-^ produced donates electrons to the membrane-bound energy-converting Ehb complex that generates a Na^+^ gradient driving ATP synthesis and regenerates H_2_ recycled for FBEB by the MvhADG:HdrABC complex. A similar FBEB/H_2_ cycling scheme is proposed for a sixth class of methanogens, ‘*Candidatus* *Methanofastidiosa*,’ based on metagenome-derived draft genomes that also lack cytochromes and genes encoding enzymes for reversal of the CO_2_ reducing pathway (Nobu et al., 2016). However, this class is restricted to reducing methylthiols with H_2_. A seventh order, the *Methanomassiliicoccales*, also grows by reducing the methyl groups of methylotrophic substrates with H_2_. Analyses of several genomes show this class also lacks cytochromes and genes required for reversal of the CO_2_ reducing pathway (Borrel et al., 2014; Kroninger et al., 2015; Lang et al., 2015). Unlike *M. stadtmanae* and the ‘*Candidatus* *Methanofastidiosa*’ class, genes encoding the membrane-bound energy-converting complexes are absent and genes encoding an Fpo-like complex and the HdrD subunit of HdrDE are present. **Figure 8** shows the pathway proposed for *Methanomassiliicoccus luminyensis.* The Fpo complex oxidizes Fdx^2-^ generated via FBEB of H_2_ by the MvhADG:HdrABC complex. HdrD accepts electrons from Fpo and reduces CoMS-SCoB coupled to generation of a H^+^ gradient. Roles for involvement of the Ech1 and Ech2 hydrogenases are ruled out based on low abundance of transcripts and low membrane-bound hydrogenase activity (Kroninger et al., 2015). Thus, CoMS-SCoB is essential for both FBEB and the terminal electron acceptor which is distinct from that proposed for *M. stadtmanae* and the ‘*Candidatus* *Methanofastidiosa*’ class which involves H_2_ cycling (**Figure 7**).

## Acetotrophic Methanogens

**FIGURE 9:**
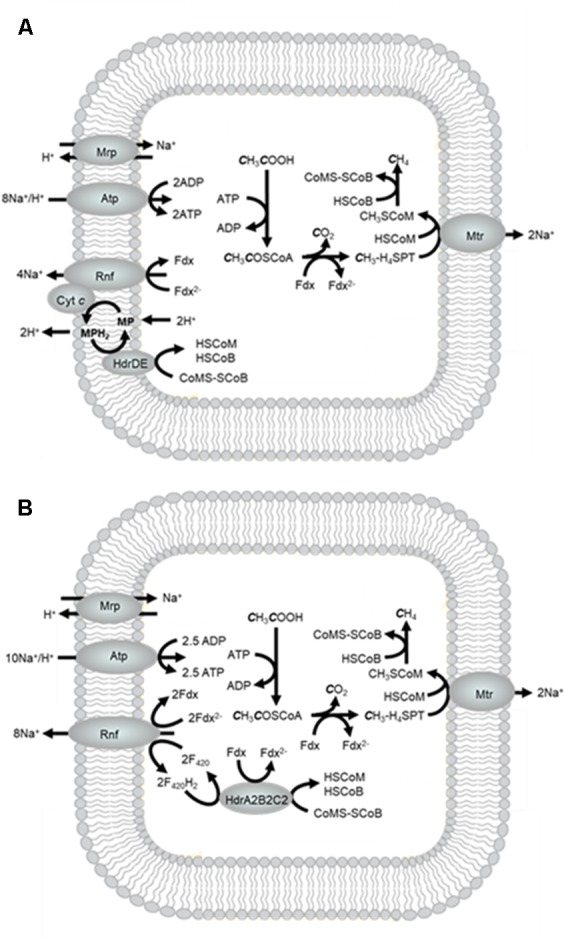
Acetotrophic pathway of *Methanosarcina acetivorans*. **(A)** Pathway based on the current biochemical understanding of electron transport. **(B)** Pathway showing electron transport based on the postulated role for FBEB by HdrA2B2C2 (Buckel and Thauer, 2018a).

*Methanosarcina* and *Methanosaeta* are the only described genera of acetotrophic methanogens that are the subject of recent reviews (Ferry, 2013; Schlegel and Muller, 2013; Welte and Deppenmeier, 2014; Ferry, 2015). Most biochemical investigations have involved *Methanosarcina* species. *M. acetivorans* is a model for species that do not metabolize H_2_ which constitute the majority of *Methanosarcina* species (**Figure 9A**). Acetate is converted to acetyl-CoA at the expense of one ATP followed by cleavage of the C-C and C-S bonds yielding a methyl group that is transferred to H_4_SPT and a carbonyl group that is oxidized to CO_2_ with transfer of electrons to Fdx. The methyl group of CH_3_-H_4_SPT is transferred to HS-CoM followed by reductive demethylation of CH_3_S-CoM to methane involving reactions common to all methanogenic pathways. The Mtr complex catalyzes the exergonic methyl transfer coupled to generation of a Na^+^ gradient. The reduced Fdx^2-^ is electron donor to the Na^+^-pumping Rnf complex that donates electrons to cytochrome *c* that is the electron donor to MP (Wang et al., 2011). As in the methylotrophic pathway, HdrDE oxidizes MPH_2_ and reduces CoMS-SCoB with scalar translocation of H^+^ that generates a gradient. The multisubunit Na^+^/H^+^ antiporter Mrp adjusts the Na^+^/H^+^ ratio optimal for the ATP synthase which is dependent on both Na^+^ and H^+^ gradients (Schlegel et al., 2012a; Jasso-Chavez et al., 2013, 2016).

When switched from growth with methanol to growth with acetate, *M. acetivorans* up regulates an electron bifurcating heterodisulfide reductase (HdrA2B2C2) that oxidizes F_420_H_2_ (*E*_m_ = -380 mV) and reduces Fdx (*E*_m_ = -520 mV) driven by reduction of CoMS-SCoB (*E*_m_ = -140 mV) (Yan et al., 2017). A role has been proposed for HdrA2B2C2 dependent on reduction of NAD-like coenzyme F_420_ (F_420_) by the Rnf complex analogous to Fdx-dependent reduction of NAD^+^ by homologous Rnf complexes from the domain *Bacteria* (**Figure 9B**; Buckel and Thauer, 2018a,b). In this way, Fdx reduced by HdrA2B2C2 is re-oxidized by Rnf thereby supplementing the translocation of Na^+^. The Na^+^ gradient formed by Rnf and Mtr could be exchanged with H^+^ by Mrp to adjust the Na^+^/H^+^ ratio optimal for ATP synthesis. The process generates more ATP than electron transport involving MP and HdrDE (**Figure 9**). However, it is reported that HdrDE is essential for acetotrophic growth suggesting the possibility of both electron transport pathways oxidizing Fdx^2-^ and reducing CoMS-SCoB (Buan and Metcalf, 2010). Having alternate electron transport pathways with different thermodynamic efficiencies could provide the cell with options for responding to fluctuations in available free energy proportional to levels of acetate in the environment. Indeed, the conversion of acetate to CH_4_ and CO_2_ provides only a marginal amount of energy available for growth (ΔG^∘^′ = -36 kJ/CH_4_) that requires cells to maximize the thermodynamic efficiency.

A genome-wide analysis of *Methanosaeta thermophila* revealed genes encoding enzymes catalyzing carbon transformation reactions in the pathway of acetate to CH_4_ similar to *Methanosarcina* species (Smith and Ingram-Smith, 2007). However, genes encoding the Rnf complex are absent in the genome of *Methanosaeta* suggesting an unknown alternative electron transport pathway and mechanism for energy conservation.

## Reverse Methanogenesis

**FIGURE 10:**
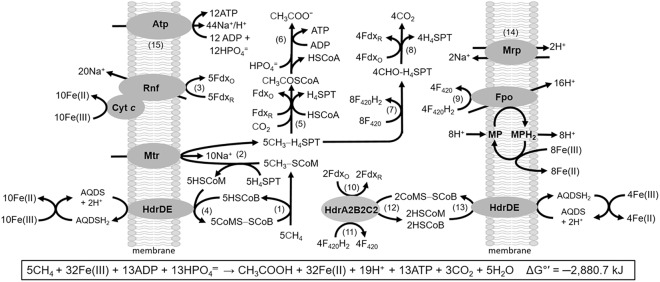
Pathway proposed for Fe(III)-dependent methane oxidation and conservation of energy by *M. acetivorans*. Enzymes not discussed in the text: CO dehydrogenase/acetyl CoA synthase (Rxn. 5); acetate kinase and phosphotransacetylase (Rxn. 6); coenzyme F_420_ (F_420_)-dependent methylene-H_4_SPT reductase, F_420_-dependent methylene-H_4_SPT dehydrogenase, methenyl-H_4_SPT cyclohydrolase, formylmethanofuran:H_4_SPT formyltransferase (Rxn. 7); formylmethanofuran dehydrogenase (Fwd) (Rxn. 8). MP, methanophenazine; AQDS, anthraquinone-2,6-disulfonate; Fdx_O_, oxidized ferredoxin (Fdx); Fdx_R_, two-electron-reduced ferredoxin (Fdx^2-^). Reproduced by permission (Yan et al., 2018).

It is postulated that AOM is accomplished by a reversal of methanogenic pathways based on environmental metagenomic and metatranscriptomic analyses of sediments (Hallam et al., 2004; McGlynn, 2017; Timmers et al., 2017). Although discovered nearly four decades ago, the unavailability of pure cultures prevented biochemical investigations of AOM. However, *M. acetivorans* is capable of “trace methane oxidation” (TMO) defined as reverse methanogenesis during net CH_4_ production from growth substrates (Moran et al., 2005, 2007; Timmers et al., 2017). More recently, methanotrophic growth dependent on reduction of Fe(III) was documented for *M. acetivorans* (Soo et al., 2016). **Figure 10** illustrates the reverse methanogenesis pathway proposed for *M. acetivorans* based on a biochemical understanding of Fe(III)-dependent mechanisms driving endergonic reactions and energy conservation essential for methanotrophic growth (Yan et al., 2018). It is remarkably similar to the pathway proposed for anaerobic methanotrophic archaea (ANME) based on metagenomic and transcriptomic analyses of uncultured *Methanosarcinales* sp. ANME-2a (Wang et al., 2014).

The *M. acetivorans* pathway is a reversal of established acetate-utilizing and CO_2_-reducing methanogenic pathways (Li et al., 2005, 2006, 2007; Lessner et al., 2006; Ferry, 2008; Wang et al., 2011; Schlegel et al., 2012b; Welte and Deppenmeier, 2014). Methane is oxidized by Mcr (Rxn. 1) with the methyl group of CH_3_-SCoM transferred to H_4_SPT by Mtr (Rxn. 2) representing the reversal of reactions common to all methanogenic pathways. The HdrDE oxidizes HSCoM and HSCoB coupled to reduction of Fe(III) that regenerates CoMS-SCoB (Rxn. 4). Removal of HSCoM, HSCoB, and CH_3_-SCoM products by HdrDE and Mtr drives the endergonic oxidation of CH_4_ by Mcr. The endergonic methyl transfer producing CH_3_-H_4_SPT is driven with the Na^+^ gradient generated by the Rnf/cytochrome *c* complex catalyzing the highly exergonic oxidation of Fdx^2-^ and reduction of Fe(III) (Rxn. 3). Fdx^2-^ is also utilized in reduction of CO_2_ that supplies the carbonyl group for condensation with the methyl group of CH_3_-H_4_SPT producing acetate (Rxns. 5 and 6). Fdx^2-^ and F_420_H_2_ are generated in reversal of the CO_2_ reduction pathway (Rxns. 7 and 8). F_420_H_2_ is oxidized by the Fpo complex (Rxn. 9) with transfer of electrons to MP and Fe(III) coupled to generation of a H^+^ gradient. The H^+^ gradient, together with the Na^+^ gradient, drives ATP synthesis assisted by the Mrp antiporter that optimizes the H^+^/Na^+^ ratio optimal for the ATP synthase dependent on both H^+^ and Na^+^ (Rxns. 14 and 15) (Schlegel et al., 2012a; Jasso-Chavez et al., 2013). The reverse methanogenesis pathway is remarkably similar to that proposed for uncultivated *Methanosarcinales* sp. ANME-2a present in marine sediments that perform AOM It is also proposed that Fdx^2-^ is generated by HdrA2B2C2 previously shown to oxidize F_420_H_2_ and reduce Fdx coupled to reduction of CoMS-SCoB via energy-conserving FBEB (Rxns. 10–12) (Yan et al., 2017). The HSCoM and HSCoB produced are oxidized by HdrDE coupled to the reduction of Fe(III) regenerating CoMS-SCoB (Rxn. 13). This proposed role would be essential in the environment where low availability of Fe(III) limits the generation of Na^+^ and H^+^ gradients by the Rnf and Fpo complexes. In this scenario, the Fdx^2-^ produced by HdrA2B2C2 is used to reduce CO_2_ for the synthesis of acetate and ATP by substrate level phosphorylation (Rxns. 5 and 6). Notably, the *Methanosarcinales* sp. ANME-2a metagenome encodes HdrA2, HdrB2, and HdrC2 homologs with 59, 72, and 59% identities (Supplementary Figure S1) consistent with a role in reverse methanogenesis by ANME.

Unlike the HdrA1B1C1 of *M. acetivorans* and the HdrABC of obligate CO_2_-reducing methanogens, the C-terminal domain of HdrA2 extends with sequences homologous to MvhD (Yan et al., 2017). Although the function of this fused MvhD is unknown, HdrA2 homologs are ubiquitous in acetotrophic and methylotrophic species of the order *Methanosarcinales* suggesting important functions. Remarkably, HdrA2 and HdrBC homologs are present in non-methanogenic species of the domain *Bacteria* signaling diverse functions.

**FIGURE 11:**
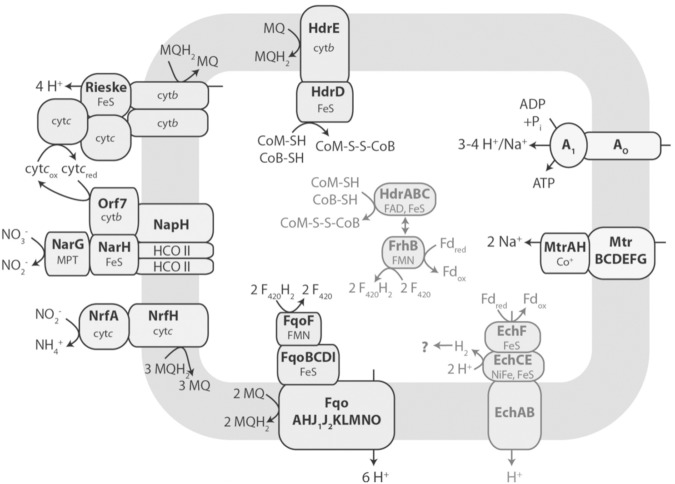
Metagenomics-based model of electron transport in reverse methanogenesis by *Methanoperedens*. Key: Fd_red_, reduced ferredoxin (Fdx^2-^); Fd_ox_, oxidized ferredoxin (Fdx); Fqo, F_420_H_2_ dehydrogenase complex; MQ, menaquinone; MQH_2,_ menaquinol; Hdr, heterodisulfide reductase; cyt *b*, cytochrome *b;* cyt*c*, cytochrome *c;* Nar, nitrate reductase complex complex; Nrf, nitrite reductase; Ech, ferredoxin-dependent hydrogenase complex; FrhB, F_420_-binding subunit of F_420_-dependent hydrogenase; Mtr, methyltransferase complex; A_1_A_O_, ATP synthase; HCO II, heme copper oxidase subunit-like proteins; FMN, flavin mononucleotide; FAD, flavin adenine dinucleotide; MPT, molybdopterin; NiFe, nickel-iron center. Reproduced by permission (Arshad et al., 2015).

A metagenomics-based metabolic model of electron transport is proposed for the nitrate-dependent reverse methanogenesis by *Methanoperedens*-like ANME (**Figure 11**). Apart from the F_420_H_2_, HSCoM/HSCoB, and Fdx^2-^ generated by reverse methanogenesis, the model contrasts with the Fe(III)-dependent pathway of *M. acetivorans* (Arshad et al., 2015). Foremost, the genome encodes a Rieske-type protein, cytochromes *c* and *b*, and a nitrate reductase that reduces nitrate to nitrite with reduced menaquinone (MQH_2_) generated by a F_420_H_2_ dehydrogenase (Fqo) that combine to generate a proton gradient driving ATP synthesis. Reduction of MQ is also accomplished by oxidation of HSCoM/HSCoB with HdrDE. An energy-converting hydrogenase homolog (Ech) is proposed to oxidize Fdx^2-^ and contribute to the proton gradient although the fate of produced H_2_ is unknown. Alternatively, Fdx^2-^ is the electron donor to a flavin-based electron confurcating complex comprised of an HdrABC homolog oxidizing HSCoM/HSCoB and donating electrons to the F_420_-dependent hydrogenase subunit (FrhB) proposed to oxidize Fdx^2-^ and reduce F_420_. Energy is conserved in the confurcation reaction rather than lost as heat should FrhB alone oxidize Fdx^2-^ and reduce F_420_ (Δ*E*^∘^′ = 120 mV). The genome of “*Candidatus Methanoperedens nitroreducens*” encodes a homolog of *M. acetivorans* HdrA2 which presents the possibility of an HdrA2B2C2 homolog catalyzing the confurcation reaction (Berger et al., 2017; Yan et al., 2017).

## Conclusion

Methanogenic and reverse methanogenic pathways are proposed to involve FBEB or FBEC in electron transport that also serve as mechanisms of energy conservation. However, there is a significant lack of understanding requiring further investigation.

(1) Biochemical confirmation of FBEB is needed for the several proposed complexes other than that shown for the purified MvhADG:HdrABC of *M. marburgensis* and HdrA2B2C2 of *M. acetivorans*.
(2) A more detailed understanding of the FBEB mechanism of HdrABC is needed. The crystal structure of MvhADG:HdrABC from *M. thermolithotrophicus* has provided a guide for experiments to address questions of electron gating and stabilization of reduced flavin intermediates. The ability to produce the catalytically active recombinant HdrA2B2C2 of *M. acetivorans*, combined with the crystal structure of MvhADG:HdrABC, provides a foundation for genetic approaches generating enzyme variants that will facilitate a detailed understanding of FBEB.
(3) Validation is needed for the proposed role of HdrA1B1C1 in the methylotrophic pathway of *M. acetivorans* and related methylotrophic methanogens; in particular, the proposal that HdrA1B1C1 of *M. acetivorans* oxidizes F_420_H_2_ in analogy to that shown for HdrA2B2C2. Also worthy of investigation are the uncharacterized HdrA2B2C2 homologs in the order *Methanosarcinales* and the domain *Bacteria*.
(4) Investigations are in order to determine the mechanism by which H_2_ is oxidized and electrons are delivered to the proposed MvhD/HdrABC and MvhD/HdrABC/Fwd complexes of methanogens in the orders *Methanocellales* and *Methanomicrobiales.*
(5) The proposed roles for FBEB and FBEC in reverse methanogenesis pathways require validation via analyses of deletion mutants.

## Author Contributions

ZY performed the research. JF wrote the manuscript.

## Conflict of Interest Statement

The authors declare that the research was conducted in the absence of any commercial or financial relationships that could be construed as a potential conflict of interest.
