# Supplementary material for: Electron Bifurcation and Confurcation in Methanogenesis and Reverse Methanogenesis
Source: Front Microbiol. 2018 Jun 20;9:1322. doi: 10.3389/fmicb.2018.01322 (PMC6019823; doi:10.3389/fmicb.2018.01322)
Supplement: Supplementary file 1 [file Presentation_1.PDF]

Supplementary Material

Figure S1. Sequence alignments of HdrA2, HdrB2 and HdrC2 from *Methanosarcina acetivorans* with HdrA2, HdrB2 and HdrC2 from the *Methanosarcinales* sp. ANME-2a metagenome. The ANME-2a genome (ID 2565956544) (https://img.jgi.doe.gov/cgi-bin/m/main.cg) was queried with protein sequences from *M. acetivorans* (https://www-ncbi-nlm-nih-gov) and aligned with CLUSTAL.

Panel A. Key: M. a., HdrA2 from *M. acetivorans* (AAM06247.1), HdrA2 from the ANME-2a metagenome (Gene ID: 2566125169). The sequences share 59% identity including four Fe_4S_4-binding motifs ($CX_2CX_2CX_3CP$) in the HdrA domain (residues 1 to 649) of HdrA2 from *M. acetivorans* denoted by (*). A motif ($CX_2CX_{25}CX_{24}CX_4C$) denoted by (#), predicted to ligate the Fe_2S_2 cluster in in the MvhD domain (residues 650-780 shown in **bold font**) from *M. acetivorans,* was found to be conserved in the MvhD domain of HdrA2 from ANME-2a (Yan et al., 2017).

Panel B. Key: M. a., HdrB2 from *M. acetivorans* (AAM07582.1); ANME-2a, HdrB2 from the ANME-2a metagenome (Gene ID: 2566124428). The sequences share 72% identity including two conserved motifs ($CX_{31–39}CCX_{35–36}CX_2C$) denoted by (*) ligating the active site [Fe_4S_4] clusters in HdrB of obligate CO_2-reducing methanogens (Wagner et al., 2017).

Panel C. Key: M. a., HdrC2 from *M. acetivorans* (AAM07581.1); ANME-2a, HdrC2 from the ANME-2a metagenome (Gene ID: 2566124427). The sequences share 59% identity including two Fe_4S_4-binding motifs ($CX_2CX_2CX_3CP$) denoted by (*).

Panel A:

```
M. a.        MRIGVYICHCGLNIAGVIDVSALEAMANELEDVVLAREVQFLCSDSGQEGIIKDIKDNKL  60
             +RIGVYICHCG NIAG +DV ++   A  L DVV+++++ + C D GQ+ I KDI++  L
ANME-2a      VRIGVYICHCGSNIAGNLDVESVREFAGNLSDVVVSKDINYACGDQGQDEIKKDIQELDL  60

M. a.        DRVVVAACSPRLHEKTFRHVMEKAGLNPYLMEMVNIREQCSWVHADDPQMATQKAFDLIR  120
             DR+VVAACSPRLHE TFR + + AGLNPYL+EMVNIREQCSWVH D    ATQKA DL+
ANME-2a      DRIVVAACSPRLHEVTFRRMAQAAGLNPYLVEMVNIREQCSWVHRDAYHYATQKAKDLVA  120

M. a.        MGVAKARFLRELSATNSKASRNVLIIGGGVAGIEAALNLAEAGFPVTMVEKESTIGGKMA  180
             MGVA+AR L  L+  +  A+R+VL+IGGGV GI+AALNLA++G  V +VE++STIGG MA
ANME-2a      MGVARARLLNPLNVESVPANRDVLVIGGGVTGIQAALNLADSGIKVHLVEQDSTIGGWMA  180

M. a.        LMNEVFPTNDCSICVLAPKMTEVQNHPNITLYTYSEVTDISGSVGKFHVRVKRKPRFVLE  240
              +N+VFPTNDCS+CVLAPKMTE  +HPNITL+TYSE+ DI G +G FHV V  KPR+V
ANME-2a      RLNDVFPTNDCSMCVLAPKMTEAADHPNITLHTYSEIQDIIGHIGNFHVSVVHKPRYVDV  240

               *  *   *   *                                    *  *  *
M. a.        DKCKGCVDLCSGVCPVEIENPMNYGIGKTRAIYMPIPQSVPQVVLIDPDHCVGCGLCQLA  300
              KCKGC++LC  VCP+E+   ++ G+   +AIY+P+ Q+VP V ++DP HCVGCGLC  A
ANME-2a      SKCKGCIELCGSVCPIEVPVEVDGGLSLRKAIYLPMSQAVPFVAVVDPKHCVGCGLCVKA  300

             *
M. a.        CPAEAVDYEQKPEEIEFEAGAIIVSTGYQLFDASRKKEYGFGKYPDVITNMQLERMLNSA  360
             C  EA+D +QK E + F+ GAIIV+TGY LFDASRK EYG+G   +VIT+M+LE+MLN++
ANME-2a      CEPEAIDIDQKEEFVVFDVGAIIVATGYHLFDASRKPEYGYGIVKNVITSMELEQMLNAS  360

M. a.        GPTGGRVLVPSTGEPPKSVAFIQCVGSRDKTVGNEYCSRVCCMAALKNSQMVKERYPDTD  420
             GPT GR++ PS G   K VAFIQCVGSRD+TVGN YCSRVCCMAA+KN+ ++  R P+
ANME-2a      GPTQGRLVRPSDGREAKRVAFIQCVGSRDETVGNPYCSRVCCMAAIKNAGLIMNRNPENH  420

M. a.        VTIHYIDIRAAGEMYEEYYTRTQEMGVDFIRGKVAEVYSGEDGRPVVRFENTLESSVEEE  480
             VT+HYIDIRA GE YEEYY + QE GV+FIRG+VA V    DG  V+ +E+T+   + +
ANME-2a      VTVHYIDIRAGGENYEEYYIKQQEKGVEFIRGRVASVKE-VDGEAVITYEDTMTGEIVDS  479

M. a.        AHDLVVLSTGYEPTKAAEGIGRMLNLARRPDRFFASAHPKMRPVDAPVSGVFLAGCASGP  540
               DLVVLS G EP   A+ I   LNL+RRPDRF   AHPKMRPVD    GVF+AGCASGP
ANME-2a      TVDLVVLSVGLEPNTNADSIVDYLNLSRRPDRFIQVAHPKMRPVDTHTRGVFVAGCASGP  539

                                                    *  *  *   *
M. a.        KEIQVSIAQGSACASKVMQLLGTGELEADPMGAHVDPDKCIGCRTCVEVCKFGKISIENK  600
             KEIQVSIAQG A +++   LL +G ++ D MG  +  + C GCR C EVC +G+I + +
ANME-2a      KEIQVSIAQGMAASARAQGLLSSGSIQKDVMGVRLVDELCNGCRLCEEVCTYGRIKVIDG  599

                     *  *  *   *                                    #  #
M. a.        KAVVDEVSCYGCGDCSAACPVGAIQMRNFENEQILAQVREATAHKSQCPFIVAFLCNWCS  660
             KAVVDE++C GCG C+AACP GA+Q R+  +EQILAQVR AT    + P I+ FLCNWCS
ANME-2a      KAVVDELTCGGCGTCAAACPRGALQTRHSTDEQILAQVRAATEDIRETPLIIGFLCNWCS  659

                                     #                        #    #
M. a.        YACADLTGMSRIRYPTNIRVIRTMCSARVNPEFVLEALKGGADGVLVAGCRMDECHYIHG  720
             YA ADL G   + YPTNIR IR MC+ RVNP FVLEALKGGADGVLVAGCR+DECHYI G
ANME-2a      YAAADLAGTQGVSYPTNIRNIRVMCAGRVNPSFVLEALKGGADGVLVAGCRLDECHYISG  719

M. a.        NFDAKKRMDILKEVIKEIGLDPKRLRTLWISAAEGERFSNTINEFVKELEEIGPIGSEFK  780
             N  A++RMD+L++V+ E+G+D  R+RT W++A+E  +F+  ++ FV++LE++G IGSE
ANME-2a      NARAQQRMDVLRDVLAELGIDQGRVRTAWVAASEAGKFAGEVSRFVEDLEKMGTIGSELD  779
```

Panel B

```
                     *                               **
M. a.       MAKLSLFRGCIVPNRYPGIEKATKLCLQKLEVDAVDLPGASCCPAPGVFKSFDKATWLAL  60
            M+KLSLF GCIVPNRYPGIEKAT+LCL KL++D  DLPGASCCPAPGVF+SFDK TWLAL
ANME-2a     MSKLSLFLGCIVPNRYPGIEKATQLCLDKLDIDCSDLPGASCCPAPGVFRSFDKPTWLAL  60

                              *  *
M. a.       ASRNIVLSERMGRDILTVCNGCYGSLADANIELKNDPEMKACTNSCLKEIGMEYKGTAEV  120
            A RNIVLSE +G D+LT+CNGCYGSL DAN ELK +   +K  TN  L +IG +Y+GT +V
ANME-2a     AGRNIVLSEELGCDVLTICNGCYGSLTDANHELKENVALKKSTNVHLGKIGKKYEGTVDV  120

                                             *
M. a.       RHIIEFLYKELGPEKLKSFITTPLDLKVALHYGCHLIKPSKERNLGETEAPVFFDELVEA  180
            RHI+EFLY E GPEK+K  +  PLDLKVALHYGCHL+KPSKER LG  E P FFDEL+EA
ANME-2a     RHIVEFLYDEFGPEKIKEMVVKPLDLKVALHYGCHLVKPSKERQLGSVEQPTFFDELIEA  180

                         **                                *    *  *
M. a.       TGAKSVDYTDKMMCCGAGGGVRSGHAAESLEMLEHKLACIRNAGVDCIVNACPFCHLQFD  240
            TGAKS+DY DKM CCGAGGGVRS     SLEM +HKL+ IR+AG DCIVNACPFCHLQFD
ANME-2a     TGAKSIDYPDKMACCGAGGGVRSALLERSLEMTKHKLSRIRDAGADCIVNACPFCHLQFD  240


M. a.       RGQLAVNEKFGTDYSIPVLHYSQLLGLALGFSPDELGIEQNAIQNIEFLAKI  292
             GQ  + EKF  +Y+IPVLHYSQLLGLALGF P ELGI+ N I N EF  K+
ANME-2a     SGQAEIREKFALEYNIPVLHYSQLLGLALGFPPVELGIDLNIITNKEFFEKL  292
```

Panel C

```
                            *  *  *   *                                *
M. a.       EELLKLLKAEGLDLLSCMHCGICTGSCPSGRHTGLNTRRIIRDARKNRAAVLSDYDLWLC  62
             ++L  L+  GLD+L+CM CG C+ SCPSGR+T LNTRRI+R     R  +L D DLW+C
ANME-2a     SDVLNTLQNAGLDVLTCMQCGTCSSSCPSGRYTSLNTRRIVRGTCVGRD-ILHDEDLWMC  62
              *  *   *
M. a.       TTCYTCQERCPRGIPITDAILELRRLAVREGLMLPEHRFVSEMVLECGHAVPLDEETKKK  122
            TTCYTCQERCPR I I D IL +R +AV EG+MLP HR VS++VL+ GHAVP++++ K+K
ANME-2a     TTCYTCQERCPREIKIADTILAIRTIAVHEGIMLPAHRKVSQLVLKYGHAVPINDDVKEK  122

M. a.       REELGLDPIPETVQKDPEALEGLKTLLKTCKFDELVA  159
            RE+L +DPIP TV K PEAL  ++TLL++C+FD+LVA
ANME-2a     REKLKMDPIPGTVHKYPEALADVQTLLRSCEFDKLVA  159
```

REFERENCES

Wagner, T., Koch, J., Ermler, U., and Shima, S. (2017). Methanogenic heterodisulfide reductase (HdrABC-MvhAGD) uses two noncubane [4Fe-4S] clusters for reduction. *Science* 357**,** 699-703. doi: 610.1126/science.aan0425.

Yan, Z., Wang, M., and Ferry, J.G. (2017). A Ferredoxin- and $F_{420}H_2$-dependent, electron-bifurcating, heterodisulfide reductase with homologs in the domains *Bacteria* and *Archaea*. *mBio* 8**,** e02285-02216. doi: 02210.01128/mBio.02285-02216.
